# An Observational Study of Telemental Care Delivery and the Context for Involuntary Commitment for Mental Health Patients in a Group of Rural Emergency Departments

**DOI:** 10.1089/tmr.2020.0005

**Published:** 2020-11-18

**Authors:** Roseanne Fairchild, Shiaw-Fen Ferng-Kuo, Hicham Rahmouni, Daniel Hardesty

**Affiliations:** ^1^ Department of Research, Richard Lugar Center for Rural Health, Union Hospital, Terre Haute, Indiana, USA.; ^2^ Department of Applied Health Sciences, Indiana State University, Terre Haute, Indiana, USA.

**Keywords:** rural emergency department, telemental, telehealth, involuntary commitment, telehealth reimbursement, suicide risk

## Abstract

**Background:** Rates for all-cause U.S. emergency department (ED) visits to rural critical access hospitals (CAHs) have increased by 50% since 2005. During the same time period, total number of U.S. hospital admissions for a mental health (MH) crisis has increased by 12.2%, with rural counties demonstrating the largest suicide rate increases overall.

**Introduction:** Increasing number of rural patients are reporting need for MH care in the region's four rural EDs. Characteristics of ED telemental health services were evaluated, including MH diagnostic category, voluntary vs. involuntary commitment (IC), forensic vs. nonforensic presentation, ED throughput, disposition, and payor reimbursement.

**Materials and Methods:** Observational 2.5-year program evaluation of telemental health care delivery for children (*n* = 114) and adults (*n* = 417) who were evaluated by a rural ED physician and received an MH diagnosis. Participants (*N* = 531) were treated by a licensed psychiatrist through telemental care delivery from September 2017 to April 2020.

**Results:** Noncommitted ED MH patients (86%; *n* = 455) were distributed across three major diagnostic groups: (1) depression, anxiety, or other mental illness (35%); (2) substance abuse (33%); or (3) suicide risk (32%), with 47% admitted inpatients (IPs), 47% referred outpatient (OPs), and 6% admitted to CAH. Fourteen percent (*n* = 76/531) of ED MH patients were subsequently IC, with 67% of those assessed as needing IP care. Forty-nine percent (*n* = 37) of IC patients presented in police custody. Most common diagnosis for IC patients was suicidal ideation/attempt (χ^2^ [2, *N* = 452] = 12.884, *p* = 0.002). Admitted patients experienced significantly longer length of stay than those with OP referral (*p* = 0.001). Mean total payor reimbursements for ED MH care were significantly lower than actual ED costs (*p* < 0.001).

**Discussion:** Innovative approaches to telemental care for IC and non-IC patients need to be piloted and comparatively evaluated in rural CAHs.

**Conclusion:** As the gateway to critically needed MH care, rural CAHs and public services pivotal to care access (e.g., law enforcement) need additional resources and support.

## Background

The number of emergency department (ED) visits for mental health (MH) care is on the rise across the United States,^1^ with rates for all-cause ED visits to small rural “safety net” hospitals reported to have increased by 50% since 2005.^2^ During this same time period, the total number of U.S. hospital admissions for an MH crisis increased by 12.2%,^3^ the rate of suicide increased >20% in 87% of U.S. counties, and rural counties demonstrated the largest suicide rate increases overall.^4^ In the midst of these challenges, ∼66% of U.S. rural and partially rural counties are designated as MH care professional shortage areas by the federal government.^5^

Hospital or residential facilities with the inpatient (IP) psychiatric beds needed for emergent MH care are scarce in rural settings.^1,6,7^ Twenty-six U.S. states have reported a shortage of IP psychiatric beds, and have growing waiting lists.^7^ Since rural critical access hospital (CAH) EDs are typically the entry point for health care in rural communities when any type of health crisis occurs,^2,8^ the current psychiatric provider shortage creates an emergent situation for rural generalist care providers who are not likely to be formally trained or licensed in psychiatric care.^1,2,8^

### The importance of telemental health in rural settings

If a psychiatric consult with an MH specialist is not immediately available, rural MH patients may be sent home to wait for days, weeks, or even months for their initial evaluation by a psychiatric specialist.^1,7–10^ The implementation of MH care delivered through telehealth, or *telemental health*, serves to provide effective and expedient access^6,7^ to psychiatric evaluation that is similar in efficacy to face-to-face care.^6, 7, 8, 11,12^ Telemental care delivery has been reported to level the playing field in rural settings, so that the major social determinants of health (ready access to care, geographic location, health insured status, and socioeconomic disparities) are addressed to foster more efficient and effective care.^8–12^ Implementation of telehealth has also been shown to decrease rural patients' ED wait time and length of stay (LOS).^8,9,11,13,14^ Current state regulations permit use of telehealth for MH services in 30 U.S. states, and for substance use disorder services in 21 U.S. states.^6(p.24)^

### Law enforcement and MH care

Law enforcement personnel are often called to assist families who are experiencing an MH crisis.^3,6,7^ Members of law enforcement are viewed as contributing to the community safety net that provides support for emergent MH situations in which someone feels endangered.^1,3,6^ As a result, police officers often bring adults or youth to hospital EDs who are judged to be a danger to self or others, and/or who are perceived as seriously deteriorating and not able to care for themselves.^3,6^ In many EDs across the United States (including the CAH EDs participating in this study), hospital administrators frequently hire off-duty law enforcement officers to provide a protective presence, and to help ED staff manage emergent MH crises.^3^

### Involuntary commitment and MH care

In some U.S. states, there is a rising number of MH patients presenting to EDs who are assessed as being a danger to themselves or others, and who are subsequently *involuntarily committed* to IP psychiatric care, or to supervised outpatient (OP) care, (assisted/assertive outpatient treatment [AOT]).^6^ Suicide-related diagnoses, including suicide attempt, suicidal ideation, and self-injury/self-harm behaviors, are a common reason underlying involuntary commitment (IC) across the United States.^6,15,16^ Other major causes of the emergent need for IC are substance abuse (SA) related, including intentional poisoning and overdose, and serious mental illnesses, including psychotic episode.^6^ In most states, the definition of “being a danger to self or others” is the primary reason for initiating IC proceedings in the ED or other clinical setting.^6,7^

### IC, ED care, and state regulations

There are processes for IC in every U.S. state, and the diagnosis of a mental illness is a required element for IC in all states.^6^ The IC process typically requires a judge's signature on the IC order, and the judge's approval usually has to occur within 48–72 h of the attending provider's initial order.^6^ Thus, judges have a pivotal role in the IC process, and can influence rates of IC in each county or region.^3,6^ For example, some judges may only agree to involuntarily commit a patient if an IP psychiatric bed is available, or if they believe in the utility of IC in general.^3,6^ States vary widely in their estimated annual rate of psychiatric commitment, ranging from 0.23 to 43.8 per 1000 persons with serious mental illness (Hawaii and Wisconsin, respectively).^17^

The administrative burden on the receiving ED for managing and processing in-house and follow-up MH care for the IC patient is substantial. In the state of Indiana, the IC process is called either “emergency detention” (nonforensic presentation; Indiana Code P.L.2-1992, SEC.20)^18^ or immediate detention (forensic presentation; Indiana Code IC 12-26-4).^18^ In both cases, if the patient needs to be held beyond 24 h, a county judge must sign the involuntary psychiatric commitment order within 72 h.

According to the 2017 Indiana State MH Survey report,^19^ 7.8 persons per 1000 (1066/137,326) with serious mental illness (adults), or serious emotional disturbance (youth), were committed to one of Indiana's six state psychiatric hospitals. The most commonly reported diagnoses for IC across the state were bipolar and mood disorders, substance use disorder, psychosis, and schizophrenia disorders.^19^ However, these state IC data do not include a subcategory for the presence of suicidal thoughts or self-harm behaviors, nor anyone involuntarily committed to a private nonstate-financed behavioral health facility.

Like most states, Indiana does not specifically report characteristics of IC cases in nonstate-financed facilities.^7^ However, a recent separate report on suicidal thoughts or behaviors that included the state of Indiana revealed that 5.23% of all adults >18 years, and 12.41% of young adults 18–25 years, reported experiencing suicidal thoughts during the past year.^20^ The latter rate for suicidal thoughts among youth was the 10th highest rate in the United States.^20^

### Growing research needs in rural telemental health care

With increasing number of patients presenting to rural EDs with serious MH issues,^1,2^ more knowledge is needed to understand the contextual issues facing rural ED providers as they deliver emergent MH care through telehealth. Experts recommend that more research is needed in telemental health to explore any differences in temporal, disposition, and cost characteristics^13^ based on different MH diagnoses.^8,14,21,22^ It is also important to highlight characteristics of MH ED patients in the context of both forensic (law enforcement) and civil or nonforensic IC cases.^3,6^ Characteristics surrounding forensic IC compared with nonforensic IC can potentially inform important MH care resource needs for rural CAHs and their communities.^3,6^

### Purpose of the study

The purpose of this study was to assess the state of telemental health care delivery for a group of rural CAH EDs in four West Central Indiana counties, in light of the context surrounding subsequent psychiatric IC or noninvoluntary commitment (non-IC) of MH patients who presented to the participating EDs. The primary variable characteristics assessed regarding ED telemental care delivery included age, gender, presentation (forensic or nonforensic), major diagnostic group, subsequent IC vs. non-IC status, disposition upon discharge (IP vs. OP), and temporal characteristics. The secondary variable characteristics evaluated were cost, primary payor, and payor reimbursement in relation to IC status, major diagnostic group, and disposition.

## Methods and Materials

### Setting and population

The Wabash Valley Rural Telehealth Network (WVRTN) serves some of the state's most economically disadvantaged and medically underserved counties in Indiana. Based on previous research studies,^9,10,23,24^ increasing number of patients and families in the targeted region are self-reporting the need for MH care. For this evaluation, characteristics that may impact telemental health care delivery in the participating rural EDs are reported for youth and adult participants of ages 6–65+ years.

The participating counties represent a predominance of county-level federal health care professional shortage areas in MH care, and a disproportionate number of individuals and families living at or below the poverty level.^25,26^ Combined socioeconomic data for the targeted region reveal that ∼21% of the population are living at or below the federal poverty level, 17% report engaging in excessive drinking and/or have been involved in substance misuse, 13% have severe housing problems or are homeless, 15% of families are food insecure, 13% of families are uninsured, and 50–95% of children in the participating counties qualify for free or reduced lunch at school.^25,26^

### Intervention, sampling, data collection, and analysis

#### Intervention

The WVRTN utilizes a care delivery model with a centralized “hub” of medical providers that delivers specialty-based psychiatric care through a regional telehealth network. For telemental care delivery, the distant board-certified psychiatric providers comprise the hub that connects to the originating rural ED provider teams. The rural ED teams are led by board-certified emergency physicians as the attending providers. For study purposes, the telemental health visit was ordered by the attending ED physician, and was initiated by the registered nurse (RN) who was managing care for the ED patients and their family.

Telemental health visits consisted of 1:1 patient–teleprovider interviews utilizing live video interaction in a private ED examination room set up for telehealth visits with the distant psychiatric specialist. The RN functioned in a facilitative “telepresenter” role, with a family member present if/as needed. Once the telemental health visit was completed, the RN supported clinical documentation and patient's discharge plan. The subsequent MH diagnosis was made by the ED physician in conjunction with the distant psychiatric specialist. Development and communication of a tailored behavioral health safety plan for the patient and family, relevant to the discharge plan, were the responsibility of the RN, in coordination with the ED care team.

#### Study sampling and informed consent

To be able to participate in the study, a telemental health visit was ordered by the attending ED physician. The facilitating nurse then explained the telemental health process to the patient and family. If the patient was a minor (child ≤17 years), the minor child was also asked to provide written or verbal assent, and the parent or guardian was asked to consent in writing for the child to be treated through telemental health. Unless a patient subsequently decided to leave against medical advice (AMA) from the ED, all patient cases involving an MH diagnosis had a telemental health visit ordered by their attending physician, which was consented to by the adult patient, or by the child and the parent/guardian.

For this study, the total population (*N* = 532) consisted of children (*n* = 115) and adults (*n* = 417) who presented to the participating rural CAH EDs either in law enforcement custody or not in law enforcement custody, and who were subsequently assessed as needing MH care. One hundred fifteen pediatric patients received an MH diagnosis from the attending rural ED physician. One hundred fourteen children were subsequently treated with a telemental health visit, with one parent/guardian and pediatric patient leaving AMA before the telemental health visit could occur. All adult MH patients, or their guardians if they had one, consented to receive a telehealth visit (*N* = 531/532; 99% participation rate).

The following list presents the three broad behavioral health diagnostic categories that were evaluated for the study, based on the Clinical Classification Software categorization available from the Agency for Healthcare Research and Quality^27^: (1) Suicidal ideation/attempt and intentional self-inflicted injury, (2) SA and overdose, and (3) anxiety, depression, and other MH disorders (other = conduct disorder, *n* = 3).

Data were analyzed using IBM SPSS version 25.0 (IBM SPSS Statistics for Windows, Armonk, NY) and statistical significance was defined as α < 0.05. Variable relationships were analyzed utilizing descriptive, parametric, and nonparametric statistical tests. The parametric statistical analyses included (1) student's *t*-test (two-tailed) to determine any statistically significant differences in telemental health characteristics for IC and non-IC participants, and for dichotomous discharge dispositions from the ED (IP vs. OP); (2) analysis of variance (ANOVA) to determine any statistically significant differences within and among the three broad MH diagnostic categories in relation to study variables; and (3) logistic regression, to evaluate risk factors for IC status and forensic status. Nonparametric statistical analyses involved chi-square tests to determine any potential relationships between and among study variables. Cases with missing data for variables of interest were not included in the analyses.

This study was approved by the regional university's institutional review board (IRB). Sampling, data, and results of statistical analyses were reported based on guidelines established by Strengthening the Reporting of Observational studies in Epidemiology statement.^28^

## Results

### Demographics

#### IC vs. non-IC comparisons

Demographic results by commitment status (IC vs. non-IC) are outlined in Table 1. A total of 531 patients who presented to participating CAH EDs were assessed as needing telemental health care. In addition, a total of 455 out of 531 MH patients in the sample were assessed as *not* needing IC. Among patients who were not involuntarily committed, there was a similar proportion of patients diagnosed across the three major diagnostic categories: (1) anxiety, depression, and other MH disorders (35%; *n* = 159); (2) suicidal ideation/attempt and intentional self-inflicted injury (33%; *n* = 151); and (3) SA related (32%; *n* = 145).

**Table 1.** Demographic Characteristics of Involuntary Commitment Versus Noninvoluntary Commitment Critical Access Hospital Emergency Department Patients

	IC	Non-IC	Chi-square test, p
	N (%)	N (%)	
Age (years)			
1–17	11 (15)	103 (23)	0.080
18–24	13 (17)	82 (18)	
25–44	34 (45)	146 (32)	
45–64	17 (22)	94 (21)	
65+	1 (1)	30 (7)	
Total	76	455	
Gender			
Female	32 (42)	230 (50)	0.179
Male	44 (58)	225 (50)	
Total	76	455	
Principle diagnosis categories			
Anxiety, mood, and other MH disorders	16 (21)	159 (35)	0.003
Suicide and intentional self-inflicted injury	40 (53)	151 (33)	
SA related	20 (26)	145 (32)	
Total	76	455	
Payors			
Medicaid	31 (47)	145 (57)	0.192
Medicare	12 (18)	47 (19)	
Private	9 (14)	33 (13)	
Self-pay/uninsured	14 (21)	29 (11)	
Total	66	254	
Disposition			
IP care	44 (69)	181 (47)	0.608
OP care	15 (23)	184 (47)	
Admit to CAH	5 (8)	23 (6)	
Total	64	388	

CAH, critical access hospital; IC, involuntary commitment; IP, inpatient; MH, mental health; OP, outpatient; SA, substance abuse.

Across all patients, there was a significantly higher number of SA cases among adults who were treated than youth ≤17 years (χ^2^ [2, *N* = 455] = 39.109, *p* < 0.001). Compared with the non-IC subsample, the number of IC patients between 25 and 44 years was disproportionately higher (*n* = 146, 32% vs. *n* = 34, 45%, respectively). The remaining IC patients assessed in the rural EDs were <25 years (*n* = 24; 32%), and 15% (*n* = 11) of IC patients were youth of ages 11–17 years.

Gender was equitably distributed among non-IC patients (50% male/50% female; *n* = 455) compared with a slightly higher number of males than females who were subsequently recommended for IC (58%; *n* = 44 vs. 42%; *n* = 32, respectively). Among non-IC patients, there was a significantly greater proportion of female youth (61%; *n* = 63) presenting to the ED for MH care than female adults (47%; *n* = 167; χ^2^ [2, *N* = 455] = 6.002, *p* = 0.014). Conversely, the proportion of non-IC male youth (39%; *n* = 40) needing MH care was lower than non-IC male adults (53%; *n* = 185). Racial ethnicity for the total number of all ED telemental health cases was ∼98.5% white, 1% black, and 0.5% Asian, which is representative of the general population in the participating counties.^25^

Regarding ED discharge disposition for all participants, a significantly higher number of IC patients were admitted IP (*n* = 44/76; 69%) than non-IC patients (*n* = 181/388; 47%; χ^2^ [2, *N* = 452] = 12.884, *p* = 0.002). Thus, fewer IC patients were set up with OP treatment than non-IC patients (23% vs. 47%, respectively). A small number of both IC (8%) and non-IC (6%) patients were admitted to ED CAH where they presented for care.

Similar to previous studies,^9,10^ the proportional majority of behavioral health patients in this study were in the Medicaid payor category, including 47% (*n* = 31) of those who were involuntarily committed and 57% (*n* = 145) of those who were not committed. In addition, a higher proportion of IC patients were uninsured than non-IC patients (21% vs. 11%, respectively). Overall, 13% (*n* = 42/320) of patients reported being uninsured.

#### IC case results: forensic vs. nonforensic

Fourteen percent (*n* = 76) of patients evaluated in this study were subsequently involuntarily committed for emergent psychiatric care. Demographic results for forensic compared with nonforensic ED IC patients are presented in Table 2. Among 76 IC patients, 49% (*n* = 37/76) presented to the ED in police custody (immediate detention) compared with 51% (*n* = 39/76) of IC patients who were not in police custody, but who were emergently treated in the ED and subsequently assessed as needing IC (emergency detention). A similar number of males (51%; *n* = 19) and females (49%; *n* = 18) were presented to the ED in police custody (immediate detention).

**Table 2.** Demographic Characteristics of Immediate and Emergency Involuntary Commitment for Emergency Department Telemental Health Patients

	Immediate IC (law enforcement custody)	Emergency IC (nonlaw enforcement custody)	p
	N (%)	N (%)	
Age (years)			
11–17	11 (30)	0 (0)	0.006
18–24	5 (14)	8 (21)	
25–44	15 (41)	19 (49)	
45–64	6 (16)	11 (28)	
65+	0 (0)	1 (3)	
Total	37	39	
Gender			
Female	18 (49)	14 (36)	0.260
Male	19 (51)	25 (64)	
Total	37	39	
Major diagnostic categories			
Anxiety, mood, and other MH disorders	6 (16)	10 (26)	0.592
Suicide and intentional self-inflicted injury	21 (57)	19 (49)	
SA related	10 (27)	10 (26)	
Total	37	39	
Payor			
Medicaid	16 (50)	15 (44)	0.701
Medicare	4 (13)	8 (24)	
Private	5 (16)	4 (12)	
Self-pay/uninsured	7 (22)	7 (21)	
Total	32	34	
Disposition			
IP care	19 (63)	25 (74)	0.658
OP care	8 (27)	7 (21)	
Admit to CAH	3 (10)	2 (6)	
Total	30	34	

The most common diagnostic category for IC patients was suicidal ideation/attempt for those experiencing both immediate (*n* = 21; 57%) and emergency detention (*n* = 19; 49%; χ^2^ [1, *N* = 76] = 1.048, *p* = 0.592). Patients experiencing suicidal ideation or attempt were 2.5 times more likely to be involuntarily committed (OR = 2.49, 95% CI [1.50–4.15], *p* < 0.001). Overall, male patients were 1.4 times more likely to be involuntarily committed (OR = 1.40, 95% CI [0.90–2.11], *p* = 0.147), and as age increased, patients were slightly more likely to be involuntarily committed (OR = 1.15, 95% CI [0.92–1.43], *p* = 0.216). Patients who were involuntarily committed were three times more likely to be transferred to IP care (OR = 3.04, 95% CI [1.53–6.04], *p* = 0.002) than to be recommended for structured AOT.

SA-related issues were reported for 26% (*n* = 20) of all IC patients. Anxiety, depression, and other MH disorders were reported among the remaining 16% of immediate detention patients (*n* = 6) and 26% of emergency detention patients (*n* = 10). Across all age groups, patients in the anxiety, depression, and other mental illness category were two times less likely to be involuntarily committed (OR = 2.03, 95% CI [1.13–3.65], *p* = 0.018) than patients at risk for suicide/self-harm.

*All youth who were IC were brought to the ED in law enforcement custody* (*n* = 11, 30%). The majority of IC youth were of high school age 14–17 years (*n* = 9, 83%), and were more likely to be female compared with the adult IC patients (χ^2^ [2, *N* = 455] = 6.002, *p* = 0.014). In all youth IC cases, a parent or guardian had called 911 for help with their child, resulting in law enforcement responding to the 911 call. Among the adult IC cases, the majority of the requests for help were initiated by the hospital ED itself, if staff needed additional help. Among adults, 34% (*n* = 26) were presented to the ED in law enforcement custody.

Similar to the larger sample of non-IC patients, Medicaid was the primary payor for the majority of immediate (50%) and emergency (44%) detention patients. In contrast, there were nearly twice as many Medicare patients who were assessed as needing emergency detention (nonforensic; 24%) compared with those presenting in police custody (immediate detention; 13%). Overall, number of uninsured patients was relatively high across the immediate (22%) and emergency (21%) detention cases.

The majority of both immediate (63%; *n* = 19) and emergency (74%; *n* = 25) detention patients were transferred and admitted to an IP psychiatric bed facility. In contrast, AOT was ordered for 27% of immediate detention and 21% of emergency detention patients. A small proportion of both immediate (10%) and emergency (6%) detention patients were admitted to the participating CAH ED where the patient presented for care. As historical context, the COVID-19 pandemic was emerging in the United States in mid-March 2020, with a very low proportion of positive cases recorded in the participating counties by the end of April 2020; the final IC case for the study period was recorded on January 13, 2020.

### Primary characteristics of telemental health

#### Temporal characteristics by IC status, diagnostic group, and disposition.

##### ED wait time (time-to-provider)

ED wait time was defined as the time from patient sign-in in the ED reception area to initiation of care by the initial assessing provider. Table 3 presents the total number of IC and non-IC MH cases by the major diagnostic group, disposition, and temporal ED variables. Both within-IC status and between-IC status group comparisons were analyzed.

**Table 3.** Temporal Outcomes of Critical Access Hospital Emergency Department Telemental Health Care Delivery

	IC		Non-IC		t* tests, *p
	n	M [95% CI]	n	M [95% CI]	
ED wait time (time to provider), min					
Total behavioral ED visits	67	19 [11 to 27]	383	23 [19 to 27]	0.420
Diagnostic within-group differences					
Anxiety, mood, and other MH disorders [A]	16	35 [3 to 66]	148	29 [20 to 38]	0.690
Suicide and intentional self-inflicted injury [B]	35	17 [10 to 23]	137	21 [18 to 25]	0.626
SA related [C]	16	7 [3 to 11]	98	17 [13 to 21]	0.405
Across diagnostic groups: ANOVA ^*^Bonferroni *post hoc* tests	*p* = 0.597		*p* = 0.053		—
	Nonsignificant		Nonsignificant		
Total behavioral ED visits	57	20 [11 to 29]	326	24 [20 to 29]	0.420
Disposition within-group differences					
IP care [A]	40	19 [6 to 32]	159	21 [16 to 25]	0.798
OP care [B]	12	19 [6 to 31]	149	28 [19 to 36]	0.556
Admit to CAH [C]	5	26 [−2 to 53]	18	29 [15 to 43]	0.805
Across dispositions: ANOVA ^*^Bonferroni *post hoc* tests	*p* = 0.920		*p* = 0.281		—
	Nonsignificant		Nonsignificant		
ED total length of stay, min					
Total behavioral ED visits	68	544 [453 to 635]	386	457 [423 to 490]	0.047
Diagnostic within-group differences					
Anxiety, mood, and other MH disorders [A]	16	543 [353 to 733]	149	412 [365 to 458]	0.092
Suicide and intentional self-inflicted injury [B]	36	520 [400 to 640]	139	458 [403 to 514]	0.326
SA related [C]	516	600 [355 to 844]	98	524 [444 to 603]	0.488
Across diagnostic groups: ANOVA ^*^Bonferroni *post hoc* tests	*p* = 0.051		*p* = 0.036		—
	Nonsignificant		*p* = 0.030 [A]:[C]^*^		
Total behavioral ED visits	58	563 [462 to 665]	329	460 [422 to 488]	0.047
Disposition within-group differences					
IP care [A]	41	612 [487 to 736]	162	431 [387 to 474]	0.001
OP care [B]	12	412 [203 to 620]	149	487 [425 to 540]	0.509
Admit to CAH [C]	5	532 [25 to 1039]	18	511 [336 to 686]	0.909
Across dispositions: ANOVA ^*^Bonferroni *post hoc* tests	*p* = 0.284		*p* = 0.266		—
	Nonsignificant		Nonsignificant		

An asterisk denotes statistical significance.

ANOVA, analysis of variance; ED, Emergency Department.

Mean wait time for IC patients was 19 min (95% CI [11–27]), with a range of 7 min (95% CI [3–11]) for SA patients to 35 min (95% CI [3–66]) for anxiety, depression, and other mental illness patients, a statistically significant difference (*p* = 0.045). Mean wait time for IC patients brought to ED by law enforcement was shorter on average than for nonforensic patients, with time-to-provider averaging 11 min (95% CI [9–24]) compared with 20 min (95% CI [6–34]), respectively. Mean wait time for non-IC patients was 23 min (95% CI [19–27]), with a range of 17 min (95% CI [13–21]) for SA patients to 29 min (95% CI [20–38]) for patients with anxiety, depression, and other mental illness.

The wait time differences based on major diagnostic group are clinically meaningful and likely reflective of the emergent care needed for SA patients due to risk for overdose, or the emergent care needed for a suicide attempt. The mean difference in wait time for forensic patients may be due to more emergent patient cases being handled by law enforcement in general, similar to other studies.^3,16^ However, no statistically significant differences in wait time were observed between IC and non-IC patients, nor between forensic (immediate detention) and nonforensic (emergency detention) IC patients.

##### ED length of stay

LOS was defined as the time from the initiation of treatment in the ED to (1) discharge from the ED or (2) transport departure time from the ED to an IP bed, if the patient was admitted. A significantly longer mean LOS was observed for IC patients who were subsequently admitted IP (9 h 23 min; *M* = 563, 95% CI [462–665 min]) than for non-IC patients (7 h 26 min; *M* = 460, 95% CI [422–488 min]; *p* = 0.047). All admitted patients (non-IC and IC) experienced a significantly longer LOS than patients discharged to OP treatment (*p* = 0.001).

The admitted IC patient's LOS reflected a mean ED stay of 10 h 12 min (*M* = 612, 95% CI [487–736 min]), whereas mean LOS for the admitted non-IC patient was 7 h 11 min (*M* = 431, 95% CI [387–474 min]). Patients who were admitted to the CAH where they presented experienced a wide-ranging ED LOS, from 25 min to 17 h 19 min. On average, IC patients spent 8 h 52 min (*M* = 532, 95% CI [25–1039 min]) in the ED before being transported to their CAH bed, similar to non-IC patients with mean ED LOS of 8 h 31 min (*M* = 511, 95% CI [336–686 min]).

There was a clinically meaningful difference observed in ED LOS between forensic and nonforensic patient cases. Patients presenting to the ED in law enforcement custody experienced an LOS of 8 h 20 min (*M* = 500, 95% CI [360–639 min]) compared with an LOS of 9 h 44 min (*M* = 584, 95% CI [460–708 min]) for nonforensic patients. Both ED wait time and ED LOS were typically shorter for forensic patients, potentially due to the perceived emergent context surrounding these cases. In contrast, for more stable ED patients who were subsequently discharged to OP treatment, the difference in mean ED LOS for IC and non-IC patients was not significant, with an LOS of 6 h 52 min (*M* = 412, 95% CI [203–620 min]) for IC patients compared with 8 h 7 min (*M* = 487, 95% CI [425–540 min]) for non-IC patients.

##### Time of IC decision to discharge from ED

The mean period of time from the attending provider's decision to involuntarily commit a patient, which included completing necessary judicial paperwork, securing a judge's approval, finding an IC bed, and completing the ED discharge, extended from 2 h 40 min (*M* = 160, 95% CI [109–210 min]) to 4 h 1 min (*M* = 241, 95% CI [−9 to 491 min]), with a total range of 2 min (internal CAH admission) to 8 h 11 min (transfer admission). On average, an IC patient waited 3 h 17 min (*M* = 197, 95% CI [121–272 min]) for ED discharge completion and transport to an IP psychiatric bed. The non-IC patient's mean ED stay was ∼3 h less than the IC patient's ED stay (7 h 11 min; *M* = 431, 95% CI [387–474 min] vs. 10 h 12 min; *M* = 612, 95% CI [487–736 min], respectively), likely a result of OP vs. IP discharge disposition.

Significant differences in ED LOS were also observed for SA patients compared with patients with depression, anxiety, or other MH disorders based on disposition. Non-IC SA patients who were subsequently admitted IP experienced significantly longer mean ED LOS of 8 h 44 min (*M* = 525, 95% CI [444–603 min]) than a mean LOS of 6 h 52 min (*M* = 412, 95% CI [365–458 min]; *p* = 0.030) for non-IC patients with anxiety, depression, or other mental illness.

The same comparison of IC patients in these two major diagnostic categories was nearly significant, with a mean LOS of 10 h (*M* = 600, 95% CI [355–844 min]) for IC SA patients compared with mean LOS of 9 h 3 min (*M* = 543, 95% CI [353–733 min]; *p* = 0.051) for IC patients with anxiety, depression, or other MH disorders. The longer LOS for SA patients is likely due to life-saving measures implemented by ED staff related to potential or actual overdose, and/or waiting for patients to “sober up” and return to conscious and alert status for a more thorough assessment before discharge decision making.

### Secondary characteristics

#### Total ED costs and reimbursement by IC status and major diagnostic group

Total mean ED costs for IC and non-IC patients have been stratified and are presented by IC status and major diagnostic group (Table 4). Mean ED costs for all SA cases were significantly higher overall for both IC (*M* = $8461, 95% CI [$6791–$10130]; *p* = 0.001) and non-IC (*M* = $8898, 95% CI [$7963–$9834]; *p* < 0.001) patients, accounting for 26% and 36% of patient cases, respectively. Patients with suicidal thoughts and/or behaviors comprised 54% of IC cases, and 36% of non-IC cases, with a mean ED cost that was lower than SA patient ED treatment (*M* = $5563, 95% CI [$4608–$6518] and *M* = $5192, 95% CI [$4616–$5769], respectively), but higher on average than costs for patients treated for anxiety, depression, and other mental illness.

**Table 4.** Total Costs of Emergency Department Visit and Percentage Reimbursement for Involuntary Commitment vs. Noninvoluntary Commitment Cases by Major Diagnostic Group

	IC		Non-IC		IC vs. Non-IC
	n	M [95% CI]	n	M [95% CI]	t* tests, *p
Total costs for ED visit					
Total behavioral ED visits	70	6130 [5368–6892]	264	6612 [6097–7127]	0.224
Diagnostic within-group differences					
Anxiety, mood, and other MH disorders [A]	14	4672 [3429–5915]	74	5449 [4510–6389]	0.488
Suicide and intentional self-inflicted injury [B]	38	5563 [4608–6518]	94	5192 [4616–5769]	0.499
SA-related [C]	18	8461 [6791–10130]	96	8898 [7963–9834]	0.702
Across diagnostic groups: ANOVA ^*^Bonferroni *post hoc* tests	*p* = 0.001		*p* < 0.001		—
	*p* = 0.001 [A]:[C]^*^		*p* < 0.001 [A]:[C]^*^		
	*p* = 0.003 [B]:[C]^*^		*p* < 0.001 [B]:[C]^*^		
Reimbursement					
Total behavioral ED visits	55	1511 [1019–2005]	231	1923 [1554–2292]	0.318
Diagnostic within-group differences					
Anxiety, mood, and other MH disorders [A]	13	1460 [392–2527]	66	1364 [889–1840]	0.870
Suicide and intentional self-inflicted injury [B]	27	977 [427–1528]	85	1316 [840–1793]	0.454
SA-related [C]	15	2519 [1292–3747]	80	2482 [1780–3184]	0.965
Across diagnostic groups: ANOVA ^*^Bonferroni *post hoc* tests	*p* = 0.024		*p* = 0.005		—
	*p* = 0.024 [B]:[C]^*^		*p* = 0.024 [A]:[C]^*^		
			*p* = 0.001 [B]:[C]^*^		
Reimbursement, %					
Total behavioral ED visits	55	24 [18–31]	231	22 [19–25]	0.619
Diagnostic within-group differences					
Anxiety, mood, and other MH disorders [A]	13	30 [14–46]	66	20 [16–25]	0.106
Suicide and intentional self-inflicted injury [B]	27	19 [10–27]	85	21 [16–26]	0.653
SA-related [C]	15	30 [14–45]	80	24 [19–29]	0.373
Across diagnostic groups: ANOVA	*p* = 0.241		*p* = 0.485		—

An asterisk denotes statistical significance.

There were significant differences in payor reimbursement based on major diagnostic group for both IC and non-IC patients. Overall, the rural CAHs received higher reimbursement amounts for SA patients in the IC (*M* = $2519, [$1292–$3747]; *p* = 0.024) and non-IC (*M* = $2482, 95% CI [$1780–$3184]; *p* = 0.005) status categories than for SA patients in both IC and non-IC patients with suicidal thoughts and/or behaviors (*M* = $977, 95% CI [$427–$1528]) and (*M* = $1316, 95% CI [$840–$1793]), respectively. In proportional terms, mean payor reimbursement percentage for SA IC and non-IC patients was at 30% (95% CI [14–45%]) and 24% (95% CI [19–29%]), respectively, whereas mean percentage reimbursement for IC and non-IC patients diagnosed with suicidal thoughts and/or behaviors was the lowest overall for the study at 19% (95% CI [10–27%]) and 21% (95% CI [16–25%]), respectively. Mean percentage reimbursement for the treatment of anxiety, depression, and other mental illness patients revealed the greatest variation between the IC and non-IC patient categories, at 30% (95% CI [14–46%]) vs. 20% (95% CI [16–25%]), respectively, a nearly significant difference (*p* = 0.106).

#### Total ED charges and reimbursement by IC status and primary payor

Total ED charges, reimbursement amount, and reimbursement percentage were stratified and are presented by IC status and primary payor (Table 5). Overall, the total billed charges for the ED visits across IC and non-IC patients were not significantly different, with a mean of $6130 (95% CI [$5334–$6926]) compared with a mean of $6637 [$6109–$7166], respectively. However, for non-IC patients, the Medicare billed amount (*M* = $9241, 95% CI [$7870–$10612]) was significantly higher than Medicaid (*M* = $5826, 95% CI [$5190–$6462]; *p* < 0.001) and private payor (*M* = $6151, 95% CI [$4909–$7393]; *p* = 0.006) categories.

**Table 5.** Total Costs of Emergency Department Visit, Total Charges, and Reimbursement Percentage for Involuntary Commitment vs. Noninvoluntary Commitment Cases by Primary Payor

	IC		Non-IC		t* tests, *p
	n	M [95% CI]	n	M [95% CI]	
Total charges for ED visit					
Total behavioral ED visits	66	6130 [5334–6926]	254	6637 [6109–7166]	0.224
Payor within-group differences					
Medicaid [A]	31	5829 [4846–6811]	145	5826 [5190–6462]	0.997
Medicare [B]	12	6900 [4230–9570]	47	9241 [7870–10612]	0.436
Private [C]	9	6744 [4512–8977]	23	6151 [4909–7393]	0.644
Self-Pay [D]	14	5742 [3539–7946]	29	7030 [5202–8859]	0.386
Across primary payers: ANOVA ^*^Bonferroni *post hoc* tests	*p* = 0.698 Nonsignificant		*p* < 0.001 *p* < 0.001 [A]:[B]^*^ p = 0.006 [B]:[C]^*^		—
Reimbursement					
Total behavioral ED visits	54	1539 [1040–2038]	231	1734 [1402–2066]	0.318
Payor within-group differences					
Medicaid [A]	28	757 [207–1309]	134	833 [545–1123]	0.825
Medicare [B]	11	1465 [460–2469]	47	2910 [1974–3847]	0.120
Private [C]	8	3608 [1690–5527]	32	2921 [2294–3548]	0.354
Self-Pay [D]	7	2416 [1424–3409]	18	3255 [1150–5360]	0.614
Across primary payers: ANOVA ^*^Bonferroni *post hoc* tests	*p* < 0.001 *p* < 0.001 [A]:[C]^*^ p = 0.027 [B]:[C]^*^		*p* < 0.001 *p* < 0.001 [A]:[B]^*^ p < 0.001 [A]:[C]^*^ p < 0.001 [A]:[D]^*^ p < 0.001 [B]:[C]^*^		—
Reimbursement, %					
Total behavioral ED visits	54	25 [18–32]	231	22 [19–25]	0.619
Payor within-group differences					
Medicaid [A]	28	10 [5–16]	134	11 [9–13]	0.987
Medicare [B]	11	19 [12–26]	47	27 [21–32]	0.177
Private [C]	8	53 [34–72]	32	50 [44–56]	0.707
Self-pay [D]	7	58 [36–80]	18	41 [29–52]	0.111
Across primary payers: ANOVA ^*^Bonferroni *post hoc* tests	*p* < 0.001 *p* < 0.001 [A]:[C]^*^ p = 0.027 [B]:[C]^*^		*p* < 0.001 *p* < 0.001 [A]:[B]^*^ p < 0.001 [A]:[C]^*^ p < 0.001 [A]:[D]^*^ p < 0.001 [B]:[C]^*^ p < 0.001 [B]:[D]^*^		

An asterisk denotes statistical significance.

Mean total reimbursement amounts across all payors were significantly lower (*p* < 0.001) than the actual ED charges for both IC (*M* = $1539, 95% CI [$1040–$2038]) and non-IC (*M* = $1734, 95% CI [$1402–$2066]) patients. In addition, Medicaid's reimbursement amounts were significantly lower than all other payors for non-IC patients (*p* < 0.01–*p* < 0.001). For IC patients, Medicaid and Medicare's reimbursement amounts were significantly lower than private payors (*p* = 0.027–*p* < 0.001).

Based on ANOVA evaluating all primary payors, Medicaid and Medicare reimbursed MH care at a significantly lower percentage of billed charges than private payors (*p* = 0.027–*p* < 0.001). For IC patients, mean reimbursement by Medicaid and Medicare ranged from 10% (95% CI [5–16%]) to 19% (95% CI [12–26%]), compared with a mean reimbursement of 53% (95% CI [34–72%]) by private payors. Slightly higher federal government reimbursement rates were observed for non-IC patients, with mean reimbursement percentage from Medicaid and Medicare ranging from 11% (95% CI [9–13%]) to 27% (95% CI [21–32%]) compared with 50% (95% CI [44–56%]) from private payors.

## Discussion

Telemental health services have been incorporated into the participating CAH EDs to increase access, responsiveness, expertise, and efficacy in the delivery of MH care to address the behavioral health care challenges in the rural communities they serve. Similar to national MH care delivery trends,^1,2,6,7^ the number of ED visits for urgent and emergent MH care is on the rise in the participating CAHs.^9,10,23,24^ These data revealed the greatest proportion (36%) of MH patients as being at risk for suicide.

### Comparative trends in suicide risk and IP hospital admissions

In the Midwestern U.S. states, including Indiana, ED visits reported for suicide risk have risen ∼34% since 2017,^29^ with comparatively higher age-adjusted suicide rates for (1) non-Hispanic whites, (2) white males, (3) nonmetropolitan (rural) populations, and (4) persons aged 35–64 years from 2005 to 2018.^29,30^ These trends are similar to those observed for this study. However, the proportion of pediatric patients at risk for suicide in this study (47%; 54/114) was observed to be 1.5 × higher than the national average of 31.6%.^20^ The proportion of patients presenting with suicide risk was even higher for patients who were subsequently involuntarily committed (49% for adults and 73% for youth), and suicide risk increased the potential for IC by 2.5 times (*p* < 0.000).

Owing, in part, to the higher proportion of patients who presented at risk for suicide, the number of hospital admissions demonstrated a robust demand for IP psychiatric beds. Nearly half of study participants (49%) were assessed as needing IP care, including 14% who were assessed as needing psychiatric commitment, and 35% who were subsequently voluntarily admitted to IP psychiatric care. However, this study characteristics revealed a lower proportion of hospital admissions among patients with suicide risk compared with an evaluation of patients treated with telehealth in a group of rural CAHs in Iowa.^15^

In the Iowa study,^15^ rural ED patients who were at risk for suicide and treated with telehealth were 2.35 × more likely to be admitted to the hospital than nontelehealth patients, with the vast majority of patients in either treatment category (telehealth, *n* = 139 and nontelehealth, *n* = 139) subsequently admitted IP (88% and 78%, respectively). As the authors point out, limiting their assessment to ED patients at risk for suicide only, rather than evaluating patients across all-cause MH ED visits, likely represented a higher level of case severity overall than all-cause MH studies.^15^

For this study, a small number of IC (8%) and non-IC (6%) patients were admitted to IP beds in the participating CAHs. In response to burgeoning MH IP needs statewide, there is currently one CAH in one county in northern Indiana not involved in this study that has incorporated 10 psychiatric IP beds into their rural safety net system. As rural CAH administrators and physicians become more accustomed to admitting behavioral health patients to an IP bed for observation and follow-up, perhaps other rural CAHs will consider seeking additional resources to (1) initiate full-time utilization of remotely based telepsychiatric specialists^31^ and/or (2) establish IP psychiatric beds and hire psychiatric physician(s) to manage these patients.

### IC: IP vs. OP care trends

The proportion of IC cases (14%) was lower in this study than in two other recent studies. In the aforementioned Iowa study, 31% of telehealth patients, and 50% of nontelehealth patients, experienced an involuntary hold for MH care.^15^ In another study of MH patients in Virginia,^31^ 2624 adults were evaluated while experiencing an MH crisis. Upon assessment by an emergency services evaluator in their community (required in Virginia), 42% of patients were described as needing IC, 31% were referred to OP treatment (non-IC), 22% agreed to voluntary hospitalization, and 5% of patients declined services.^31^

Per McGarvey and colleagues,^31^ AOT was rarely used in place of IP IC in Virginia. Similarly, for this study, fewer IC patients were set up with OP treatment than with non-IC patients (23% vs. 47%, respectively). However, significantly improved MH outcomes have been observed with IC patients through a structured OP treatment program.^32^ In a 4-year longitudinal randomized controlled trial Schöttle et al.^32^ reported a 25% reduction in IC for seriously mentally ill patients (*N* = 171) through utilization of a structured OP MH treatment program (assertive community treatment [ACT]). The piloted ACT program was an OP extension of the hospital setting.

According to the authors, owing to the study's availability of ACT, the MH participants who had avoided or had never experienced the sometimes “traumatic” nature of IP IC demonstrated significantly improved severity of illness (*p* = 0.004) and functional status (*p* = 0.043) over time.^32^ The majority (69.2%) of patients were also fully adherent to their OP treatment plan at 4-year follow-up (*p* < 0.001).^32^ Based on results of this study, implementation of a longitudinal study of adult and pediatric patients identified early in the rural communities as at risk for suicide could proactively serve to further inform participating rural stakeholder groups in the evaluation of targeted evidence-based structured MH OP treatment services.

### Reimbursement trends in telemental health care

Telehealth-based care, in particular, has been utilized primarily for MH services compared with all health care services in the study region.^9,10,24^ Increased utilization of telehealth to treat MH patients has also been reported nationwide, per recent Medicaid (93%) and Medicare (70%) claims data studies.^33,34^ In contrast to increased utilization of telemental health, and the requisite efficacy and efficiencies reported to accompany it,^1,6–15,24^ reimbursement percentages for telemental care services by Medicaid and Medicare in this study were remarkably low, similar to previous regional studies.^9,10,24^ Mean total payor reimbursement amounts for both IC and non-IC patients were significantly lower than actual ED costs, whether disposition from the ED was to IP or OP care (*p* < 0.001) (Table 5). Reimbursement percentage from Medicaid was also significantly lower than private payors for both IP and OP cases (*p* < 0.001).

Experts tell us that attending rural ED providers, in conjunction with their telemental care provider partners, are highly impactful in MH assessment and treatment.^8,15^ ED providers must manage the complex administrative burden of setting up MH IP and/or OP placement and MH follow-up care with a very small ED staff,^8,15^ typically comprising one physician and two nurses for this study. Even for the most common and sometimes fatal MH crisis observed in the participating CAHs, suicidal ideation/attempt, mean total payor reimbursement ranked the lowest among all reimbursement amounts (Table 4).

Low telehealth reimbursement rates, including lower rates for rural telehealth “presenter” networks for behavioral health patients covered by Medicaid, have been reported in several studies.^33,35–37^ However, Centers for Medicare and Medicaid (CMS) recently announced increases in reimbursement rates for telehealth care delivery by physicians for calendar year 2021.^38^ Increased CMS reimbursement levels for telehealth should enhance the financial margins for rural U.S. CAHs currently utilizing telehealth in their rural health care systems.

As MH care provider shortages abound across the country,^5^ and due to variable Medicaid and Medicare reimbursement based on MH provider type,^33,35–37^ another important reimbursement issue is provider-specific telehealth reimbursement. In some U.S. states, licensed MH therapists, neuropsychologists, social workers, and MH counselors are only reimbursed for in-person care, but not care delivered through telehealth.^33^ Community stakeholders and legislative policy makers need to be made aware of the critical nature of the public policy changes that would be needed to support expansion of provider-specific reimbursement plans, to promote increased utilization of telemental health in general.^33^ Enhanced nonphysician provider reimbursement was not addressed by CMS in the aforementioned telehealth reimbursement changes for 2021.^38^

### Telemental care delivery and telehealth parity laws

MH care experts have recently recommended creation of a robust evidence-driven continuum of care across the life span, extending from community-based OP MH services to psychiatric IP services.^1,3^ To support these initiatives in MH provider shortage areas, the introduction and implementation of telehealth parity laws would bolster all categories of payor reimbursement for telemental health.^33,34^ Comprehensive statewide telehealth parity laws requiring payors (public and private) to provide the same reimbursement amounts for telehealth care delivery compared with in-person care would allow health care administrators to seamlessly integrate all available licensed MH care professionals to collaboratively address community-level MH care needs.^33^

### Limitations

This study has several limitations, and results should be interpreted cautiously. Utilization of an observational study design for enrolling participants in the study meant that all MH patients presenting to the rural EDs were able to access telemental care, allowing for no case control to be observed concurrently, to establish a “telehealth vs. nontelehealth” controlled comparison. This approach was preferred by IRB reviewers, so that all rural MH patients had access to specialist-based psychiatric care in the CAHs. Although the authors believe it is important to note and discuss emerging MH trends for children and adults in this regional study, results are not necessarily generalizable to other regions or groups of rural hospitals.

## Conclusions

The incidence of suicide risk was high for this study, especially among youth. In addition, all youth who were involuntarily committed (IC) were brought to the ED in law enforcement custody, and were more likely to be female compared with the adult IC patients, who were more likely to be male. The likelihood of IC was highest for the suicide risk group, underscoring the need for immediate and intensive psychiatric care through telemental health in rural settings when someone is a clear danger to themselves or others.

As a result of the prevalence of suicide risk for both youth and adults in this study, OP care was less likely to be recommended at ED discharge, and ED LOS was fairly long for those needing IP admission, similar to other studies,^39,40^ especially in IC cases.^31,32^ However, patients who presented in police custody experienced somewhat shorter ED wait times and ED LOS, potentially reflecting providers' perceptions of the need for emergent intervention.^31,41^ More research is needed to determine the relative effectiveness regarding evidence-based MH treatment and preventive care activities through telemental health compared with nontelemental health among youth and adults in the region.

Most rural U.S. counties, including those in West Central Indiana, do not have community MH “evaluators” (i.e., Virginia), nor community MH centers with structured OP programs. However, owing to the increased incidence of MH cases overall, these CAHs typically had off-duty law enforcement officers on staff during busy times in their rural EDs. As Pinals and Fuller point out, “Law enforcement and families rely on the Emergency Departments of their local hospitals for psychiatric crisis intervention … and the demand for competency restoration in state hospitals continues to grow…. Boarding [waiting for psych beds in EDs] is a symptom of need and resources that are not balanced.”^3(pp.14–15)^

To this end, MH experts recently recommended broad-based implementation of the following: (1) the general public's access to, and education in, preventative MH care across the life span; (2) community ACT (supervised OP) programs; (3) MH training for law enforcement; (4) real-time information regarding IP psychiatric bed availability for EDs/hospitals in each state; (5) evaluation of current state laws and criteria governing IC; (6) increases in MH workforce funding; and (7) increases in Medicaid reimbursement.^3^

Experienced ED clinicians and administrators tell us that the social and community challenges that MH patients face on a daily basis frequently impact ED throughput and resources, but also extend beyond the ED setting out into the communities.^42^ Moreover, administrative, clinical, and patient/provider needs for EDs must be anticipated, monitored, and supported, especially in resource-poor rural hospitals.^42^ As the gateway to critically needed MH care for rural residents,^2,8^ attention must be paid to the need for more comprehensive community-wide MH services,^42^ including the explicit recommendation to “incentivize and reward the use of technology [telehealth] to advance care delivery, promote appropriate information sharing, and maximize continuity of care.”^3(p.2)^

## Abbreviations Used

ACT: assertive community treatment
AMA: against medical advice
ANOVA: analysis of variance
AOT: assistive outpatient treatment
CAH: critical access hospital.
CMS: Centers for Medicare and Medicaid
ED: emergency department
IC: involuntary commitment
IP: inpatient
IRB: institutional review board
LOS: length of stay
MH: mental health
OP: outpatient
RN: registered nurse
SA: substance abuse
WVRTN: Wabash Valley Rural Telehealth Network
